# Model of Stratified Oral Health Literacy (MELSO): Integrating Social Determinants, Education, and Health Outcomes

**DOI:** 10.3390/healthcare14213569

**Published:** 2026-10-22

**Authors:** Nélio Veiga, Patrícia Couto, Helder Costa, Joana Janeiro, Sara Lopes, Iolanda Sousa Azevedo, Pedro C. Lopes

**Affiliations:** 1 Faculty of Dental Medicine, Catolica University Portugal, Estrada da Circunvalação, 3504-505 Viseu, Portugal; pscouto@ucp.pt (P.C.); hecosta@ucp.pt (H.C.); jjaneiro@ucp.pt (J.J.); snlopes@ucp.pt (S.L.); iosousa@ucp.pt (I.S.A.); paflopes@ucp.pt (P.C.L.); 2 Center for Interdisciplinary Research in Health (CIIS), Catolica University Portugal, 3504-505 Viseu, Portugal

**Keywords:** oral health literacy, social determinants of health, health education, socioeconomic inequalities, conceptual framework

## Abstract

Background/Objectives: Oral health literacy (OHL) and oral health outcomes are socially patterned. This article proposes the Model of Stratified Oral Health Literacy (MELSO) to specify how educational resources and service conditions may shape the development and use of OHL. Methods: A narrative conceptual synthesis integrated health literacy theory, social determinants frameworks, and educational perspectives into five interrelated levels. No new participant data were collected or analysed. Results: MELSO links structural context, educational and cultural capital, functional, interactive, and critical OHL, self-management behaviours, and oral health outcomes. Its proposed added value is the joint specification of educational mediation, access-related moderation, and competency-specific associations within oral health. Five testable hypotheses and a staged strategy for measurement development and empirical evaluation are presented, including comparisons with simpler models. Conclusions: MELSO is a hypothesis-generating framework rather than a validated explanatory model or measurement instrument. Its utility depends on empirical evidence that the proposed relationships are distinguishable, reproducible, and informative beyond existing approaches.

## 1. Introduction

The concept of health literacy has evolved from a focus on reading skills towards cognitive, communicative, and practical competencies. Nutbeam distinguishes functional, interactive, and critical health literacy [1]. MELSO adopts these as interrelated dimensions of competence rather than fixed stages through which every person must pass.

Functional literacy concerns understanding information and instructions; interactive literacy concerns communication and the use of information in changing situations; critical literacy concerns appraisal and informed action, including attention to social conditions [1,2]. Although these dimensions may involve different demands, their coexistence does not establish a universal developmental hierarchy.

Subsequent integrative models further refined this conceptualisation. Sørensen et al. (2012) proposed a comprehensive framework defining health literacy as the ability to access, understand, appraise, and apply health information across healthcare, disease prevention, and health promotion contexts [3]. This approach explicitly positions health literacy within social systems and emphasises its relevance for empowerment and equity.

Within dentistry, oral health literacy has been associated with oral health behaviours and outcomes [4,5,6,7]. Early instruments, such as the Rapid Estimate of Adult Literacy in Dentistry (REALD-30) and the Oral Health Literacy Instrument (OHLI), emphasise word recognition, comprehension, and numeracy [8,9], whereas multidimensional measures, such as the Health Literacy in Dentistry scale (HeLD), extend assessment beyond functional skills [9]. The issue addressed here is therefore how distinct competencies and contextual conditions can be specified together in an empirically assessable explanatory model.

### 1.1. Social Determinants of Health and Oral Health Inequalities

A substantial body of evidence demonstrates that health outcomes are socially patterned and shaped by structural conditions rather than individual choices alone. The social determinants of health framework proposed by Göran Dahlgren and Margaret Whitehead remains a cornerstone of research on health inequalities. This model conceptualises determinants as concentric layers, ranging from individual characteristics to broader socioeconomic, cultural, and environmental contexts [5].

According to this framework, individual behaviours are embedded within social networks, living and working conditions, and macro-level determinants such as economic policies, educational systems, welfare arrangements, and healthcare organisation (Figure 1). Oral health outcomes closely follow these social gradients. Disadvantaged populations consistently exhibit a higher prevalence of dental caries, periodontal disease, tooth loss, and unmet dental needs, regardless of overall improvements in dental technology and clinical care [10].

Frameworks of social determinants establish the importance of structural conditions, while integrated health literacy models already connect societal and individual antecedents with health behaviours and outcomes [3,11,12]. For oral health research, a useful further step is to specify which educational resources may translate socioeconomic differences into differences in OHL competencies and which service conditions may enable or constrain their use. These mechanisms require explicit hypotheses rather than an assumption that OHL alone explains oral health inequalities.

Access to dental care interacts with socioeconomic position (SEP) to influence both exposure to oral health information and opportunities for learning. Consequently, oral health literacy should be understood as a socially produced competence that reflects cumulative exposure to educational resources, supportive environments, and institutional structures [13].

### 1.2. Education, Cultural Capital, and Competency Development

Research on education and health supports the consideration of learning resources beyond formal educational attainment [14,15]. Within MELSO, schooling, general literacy, and household learning opportunities are proposed as resources for acquiring and using oral health competencies; their specific contributions require empirical testing.

The educational component is informed by contextual learning and ecological perspectives rather than a single stage-based learning theory. Family practices, dialogue, school experiences, and encounters with services may offer different opportunities to develop competencies [16]. MELSO does not assume that acquiring functional skills necessarily precedes all interactive or critical abilities.

General literacy, numeracy, and digital literacy are treated here as educational resources that may support oral health information use. Their distribution and relationship to OHL should be examined rather than inferred from years of schooling. General digital literacy is distinguished from health-specific digital literacy in Section 3.2 [17,18].

Across the life course, learning opportunities may change through experiences within families, schools, workplaces, communities, and dental care settings [16,19]. This provides a rationale for a temporal dimension in MELSO, but does not establish a validated account of age-specific development or an inevitable progression towards a particular literacy profile.

### 1.3. Conceptual Gaps in Existing Oral Health Models

Integrated health literacy models already relate competencies to social and personal antecedents, healthcare use, and outcomes [3,19,20,21]. The question for MELSO is therefore how to specify and test educational mediation and service-related moderation jointly in oral health, rather than whether social context has previously been recognised. The narrative synthesis does not establish the absence of comparable frameworks.

This specification addresses two research questions: whether educational resources and distinct OHL competencies account for part of the socioeconomic gradient in oral health, and whether the association between OHL and subsequent preventive action differs according to access to supportive dental services. These questions distinguish the social production of competencies from the opportunities to use them.

Accordingly, this article presents MELSO as a theory-informed, hypothesis-generating framework. Its objectives are to clarify its relationship to established models, specify testable pathways and the findings that would challenge them, and outline a measurement and validation strategy. The five levels organise explanatory constructs; they are not validated patient categories or a universal sequence of literacy attainment (Figure 2).

## 2. Materials and Methods

MELSO is developed through a narrative conceptual synthesis of three families of theoretical perspectives rather than a systematic synthesis of evidence. Nutbeam’s framework distinguishes functional, interactive, and critical competence [1,2]; Dahlgren and Whitehead offer a layered account of structural and contextual determinants [5]; and educational and ecological perspectives inform the consideration of learning resources and the settings in which competencies develop [14,15,16]. The third family comprises related perspectives rather than a single unified theory. Selection is purposive: each family addresses a different explanatory question relevant to the proposed oral health pathways.

The comparison in this revised synthesis is guided by four explicit questions: which construct is defined, at what contextual or individual level it operates, what explanatory role it could play, and what it leaves unspecified for oral health research. Table 1 records the resulting mapping. Here, complementary means addressing different parts of the proposed explanation without treating the source theories as interchangeable or their integration as proof of compatibility. Overlapping concepts are separated by role: general learning resources are candidate antecedents, OHL dimensions are competencies, and service conditions may alter opportunities for action. Potential incompatibilities and uncertain construct boundaries remain matters for empirical evaluation.

The use of literature is selective and theory-informed. Foundational works in the bibliography underpin the three families of perspectives; integrated models and models of causal pathways inform the comparison [3,19,21], and instrument-development studies inform measurement [8,9,17,18,19,20,21,22,23,24]. A targeted search to verify sources was undertaken for this revision on 17 October 2026, initially using web searches restricted to PubMed records and subsequently using exact titles or identifiers to locate publisher pages and available full texts« This targeted search is not exhaustive and does not reconstruct the original literature search, for which no search strategy was documented. No systematic screening, independent duplicate extraction, or formal grading of the evidence was undertaken. No new participant data were collected or analysed.

In this synthesis, ecological refers to interactions among people and their family, school, community, service, and policy environments, including the everyday cultural practices through which learning occurs [16,23]. It does not refer to environmental sustainability or simply to the assignment of area-level exposures to individuals. The comparison therefore treats learning as situated in social interactions and treats area or service conditions as potential contextual influences. This perspective motivates multilevel and repeated-measures designs, but does not establish that these influences are causal.

**Table 1:** Comparative mapping of the source perspectives.

Source Family	Explanatory Role and Boundary	MELSO Specification
Health literacy [1,2,3,19]	Defines competency types and information-processing activities; these are not equivalent classifications.	L comprises functional, interactive, and critical dimensions; access, understanding, appraisal, and application may draw on several dimensions.
Social determinants [5,13]	Locates socioeconomic and policy conditions; contextual association alone does not establish mediation.	S is defined independently of educational mediator E. External access A and care quality Q have named downstream moderation roles.
Education and ecology [14,15,16,23]	Relates learning resources and social settings to opportunities for competency development; does not establish fixed literacy stages.	E is a potential mediator of S–L. Changing resources and repeated care experiences motivate a life-course hypothesis.

## 3. Results: The MELSO Framework

MELSO is a socioeducational conceptual framework proposing how OHL may develop and vary across socioeconomic and educational circumstances and how it may relate to behaviours and oral health outcomes. The five levels organise constructs with different explanatory roles; they do not represent a temporal sequence. All proposed relationships, including the temporal and feedback mechanisms, await empirical validation.

The framework distinguishes structural context, educational and cultural resources, OHL dimensions, behaviours, and outcomes. Competencies may change through repeated learning and care experiences across the life course, while service conditions may affect whether they can be used. Figure 3 shows the architecture, access and care quality as candidate moderators, and possible feedback; Figure 4 separates the core forward hypotheses from later reciprocal relationships. These diagrams specify research questions rather than established causal dynamics.

**Figure 4:**
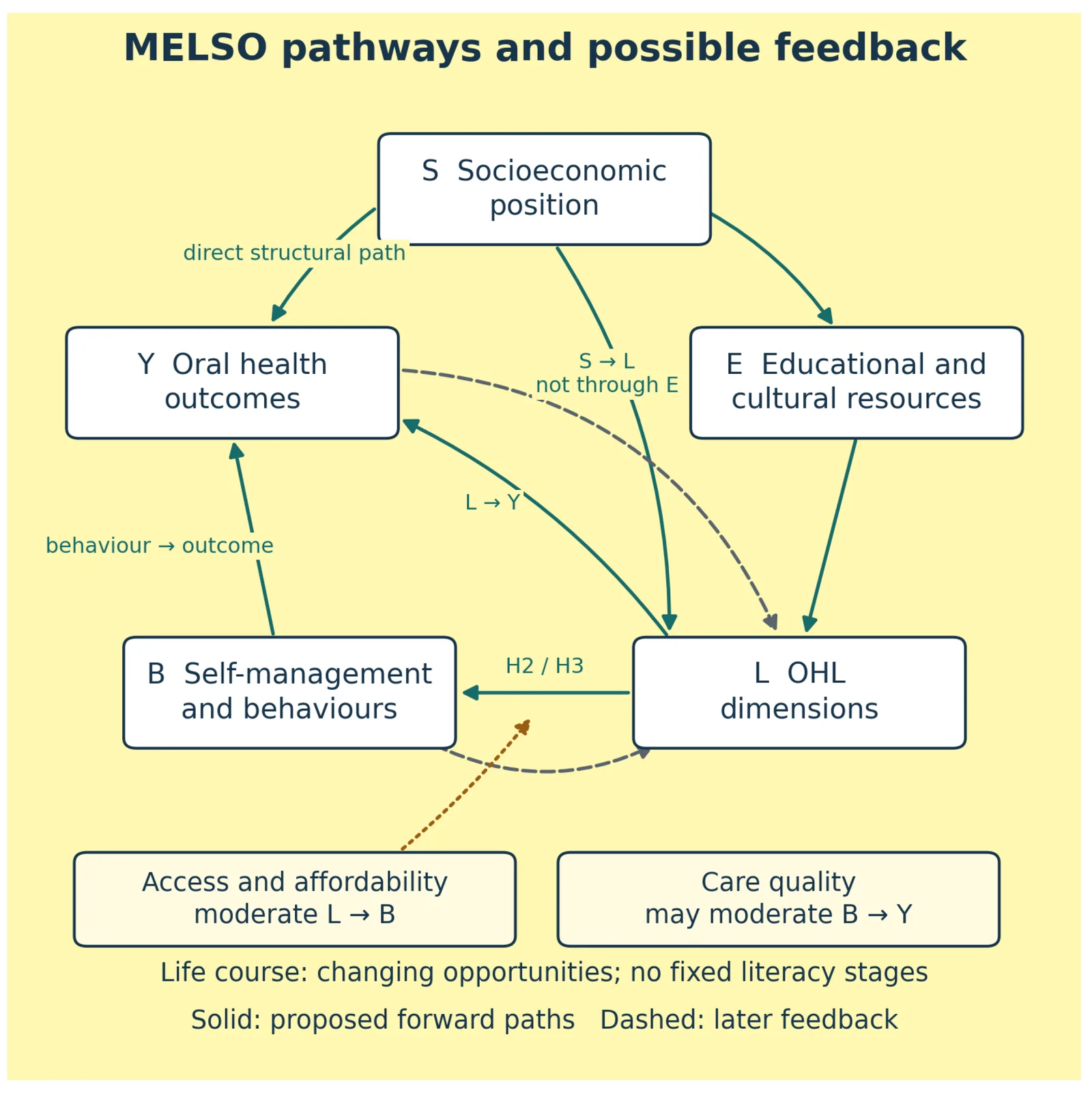
Circular presentation of hypothesised MELSO pathways and later feedback. S, E, L, B, and Y identify the constructs defined in Section 3.6 and Table 2. Solid arrows show proposed forward paths, including B to Y and S to Y; the S to L and L to Y paths allow partial mediation. Access is proposed to moderate L to B, while care quality may moderate B to Y. Dashed arrows show possible later feedback from B and Y to L; they do not imply that a contemporaneous circular causal model has been validated. Self-efficacy and social support are optional extensions. Placement around the circle does not represent developmental stages. The yellow background identifies the revised figure.

**Table 2:** Proposed pathway roles and analytical boundaries.

Relationship	Specified Role	Interpretation for Testing
S → E → L	E is a potential mediator; S is the exposure; L is the outcome.	H1 tests an indirect S–L association through E, with S defined independently of education.
E → L → B	L is a potential mediator; E is the exposure; B is the outcome.	Separate from H1; requires E, L, and later B with appropriate temporal ordering.
L → B → Y	B is a potential mediator; L is the exposure; Y is the outcome.	H4 tests an indirect L–Y relationship through preventive B; include a direct L–Y path.
S → L; S → Y; B → Y	Additional structural and behavioural paths.	Direct means relative to included mediators; these paths do not demonstrate an unmediated causal effect.
A modifies L → B	A is external access: availability and affordability.	H3 specifies L-by-A moderation; realised attendance remains B, not A.
Q modifies B → Y	Q is independently defined care quality.	Candidate B-by-Q interaction, requiring an outcome-specific hypothesis beyond H1–H5.
Optional extensions	Self-efficacy: L–B mediator; social support: L–B moderator.	Prespecify separate hypotheses and measures; these are not defined as OHL dimensions.
Earlier B or Y → later L	Potential feedback over time.	Repeated measures may test learning from care or disease experience; no contemporaneous causal loop is assumed.

### 3.1. Structural Context (Macro-Level Determinants)

The first level of the MELSO framework encompasses structural and contextual determinants that shape the conditions under which oral health literacy can develop. These include socioeconomic position (income, occupation, and employment security), public oral health policies, availability and organisation of dental services, financing mechanisms, and territorial inequalities in access to care [5,13].

The proposed indirect structural pathway operates through educational and cultural resources. A structural association with OHL not mediated by these resources is also plausible: policies requiring accessible information, the availability of interpreters, and routine opportunities to receive explanations during dental encounters may affect the comprehension or use of oral health information among people with comparable measured educational resources [19,25,26]. Such a path is direct only relative to the educational mediator included in a given analysis; it may involve unmeasured learning or organisational mechanisms. Service access should not be equated with OHL itself, and the residual association must not be interpreted as an established direct causal effect.

Unfavourable structural conditions may constrain opportunities for OHL development even when individuals are motivated to learn. Across childhood, adolescence, and adulthood, changes in schooling, household resources, employment, and dental care experiences may alter these opportunities. This life-course proposition does not specify validated stages or imply that structural disadvantage determines an individual’s competency profile [16,19].

Accordingly, future empirical applications of MELSO should operationalise the structural context using both individual-level indicators of SEP and area-level measures (e.g., service density, public coverage, territorial deprivation indices), ideally within multilevel analytical designs capable of disentangling compositional and contextual effects.

### 3.2. Educational and Cultural Capital

Level 2 comprises educational and cultural resources hypothesised to mediate part of the relationship between socioeconomic position and subsequent OHL. For H1, the independent variable (S) is defined using income, occupation, or area deprivation; the candidate mediator (E) comprises separately defined indicators of schooling, general literacy and numeracy, general digital skills, and household learning resources; and the outcome (L) is multidimensional OHL. Educational attainment must not simultaneously define S and E. A specific study should prespecify whether E is a set of separate mediators or a justified composite, rather than assuming a single reflective construct [14,15,16].

General digital literacy is defined here as the ability to operate and navigate digital tools across everyday settings and is included among the educational resources represented by E. Digital health literacy concerns finding, understanding, appraising, and using health information or services through digital media [17,18]. The Digital Health Literacy Instrument (DHLI) and the eHealth Literacy Scale (eHEALS) should therefore not automatically replace a measure of general digital skills. If health-specific digital competencies are included, they should be modelled separately and mapped to L when they are specific to oral health, with overlap assessed before scores are combined.

Education is not synonymous with years of schooling. Household learning materials, family practices, and community learning opportunities may contribute beyond formal attainment [14,15,16]. These resources should be distinguished from the person’s demonstrated oral health competencies to avoid defining the mediator and outcome with the same items.

Candidate E indicators include standardised educational attainment, general literacy and numeracy tasks, general digital navigation tasks, and defined household learning resources. Their proposed mediating role should be evaluated by measuring E before subsequent L and accounting for baseline OHL and relevant confounding. The subsequent E–behaviour relationship may also be partly mediated by L; this is a separate estimand specified in Section 3.6.

### 3.3. Oral Health Literacy (Core Construct)

For MELSO, OHL is defined as the context-dependent capacity to obtain, understand, appraise, communicate about, and use oral health information and services to make informed decisions and support individual or collective oral health. This working definition adapts established health literacy concepts to the oral health domain [1,2,3,19]. Within framework level 3, functional, interactive, and critical literacy are dimensions, not ranked patient categories:

- Functional oral health literacy refers to the ability to understand basic oral health information and follow simple instructions related to oral hygiene practices, fluoride use, and dental appointments.
- Interactive oral health literacy encompasses skills that enable individuals to apply information in daily life, communicate effectively with dental professionals, and adapt behaviours to different contexts.
- Critical oral health literacy involves the capacity to evaluate the quality and relevance of oral health information, question recommendations, weigh treatment options, and make informed decisions aligned with personal and social circumstances.

The three dimensions describe types of competence, whereas accessing, understanding, appraising, and applying describe information-processing activities [1,2,3]. These classifications are related but do not correspond one-to-one. For example, interpreting written instructions draws on functional competence; seeking clarification of those instructions and discussing how to apply them draw on interactive competence; and evaluating a source or discussing alternatives draws on critical competence. Each activity may draw on more than one dimension. People may have uneven profiles, and changing contexts may support or challenge any dimension without a fixed progression.

MELSO adopts, rather than introduces, the multidimensional understanding of health literacy [1,2,3,19]. In oral health, instruments such as HeLD already recognise domains beyond functional skills [24]. MELSO uses these established perspectives to ask whether competency profiles have explanatory value within the proposed socioeducational pathways, beyond that provided by schooling or functional literacy alone.

MELSO does not commit to a single instrument or require a new scale a priori. Initial studies may use measures validated for the relevant population and language, such as REALD-30 or OHLI for selected functional indicators, and HeLD for broader self-reported capacities [8,9,24]. Table 3 specifies their limits. A new or adapted measure is warranted only if construct mapping identifies important gaps, particularly in the assessment of critical appraisal or interactive performance; its content, dimensionality, reliability, and validity would then require testing as described in Section Measurement Development and Validation.

### 3.4. Self-Management and Oral Health Behaviours

The fourth level represents self-management and oral health behaviours, which are hypothesised to mediate part of the relationship between OHL and later outcomes. Candidate behaviours include oral hygiene practices, fluoride use, sugar consumption, preventive attendance, and adherence to agreed preventive or therapeutic plans.

OHL may influence self-management through comprehension, communication, and confidence in using information, but does not determine behaviour on its own [4,21]. The core model proposes that access, including availability and affordability, moderates the OHL–behaviour relationship. Self-efficacy is a candidate additional mediator between OHL and behaviour, and social support is a candidate additional moderator; neither is assumed to be an OHL dimension. These optional extensions are distinguished from the core hypotheses in Section 3.6.

Consequently, similar OHL profiles may be associated with different practices when affordability, service availability, social support, or competing demands differ. This is a proposed contextual explanation of heterogeneity, not a finding established by MELSO.

Candidate measures include validated self-reports of oral health practices, adherence indicators, and service records. Repeated assessment would help distinguish sustained preventive practices from short-term responses and preventive attendance from symptom-driven visits.

### 3.5. Oral Health Outcomes

Framework level 5 groups oral health outcomes for analytical purposes; it is not a final temporal stage. Clinical indicators may include incident caries, periodontal status, tooth loss, and pain, while patient-reported indicators include oral health-related quality of life [10,20]. Disease and treatment experiences may subsequently influence information seeking, OHL, and behaviour, so outcomes at one time point may become determinants at a later time point.

The core specification includes a proposed behaviour-to-outcome path and a structural-to-outcome path not transmitted through the measured literacy–behaviour sequence. For example, service coverage and the provision of preventive care may affect disease independently of individual OHL. These paths allow partial rather than complete mediation and preserve the possibility of social gradients that literacy interventions alone cannot remove [11,12,13].

Clinical examinations and repeated patient-reported measures should be selected for the population and follow-up interval. The Oral Health Impact Profile (OHIP) is an example for adults; the Child Perceptions Questionnaire for ages 11–14 (CPQ11–14) is an example for children and young adolescents [27]. Younger age groups require measures validated for their age, language, and reporting method; an instrument developed for adults should not be applied automatically to younger age groups. Baseline disease and age-appropriate outcomes are needed to distinguish accumulated burden from changes during follow-up.

### 3.6. Hypothesised Pathways: Mediation and Moderation

The core mediation specification follows the pathway S → E → L → B → Y: socioeconomic position (S), educational and cultural resources (E), OHL dimensions (L), behaviours (B), and oral health outcomes (Y). E is hypothesised to mediate part of S–L; L may mediate part of E–B; and B may mediate part of L–Y. These are separate indirect relationships with different exposures, mediators, and outcomes. Table 2 makes their roles explicit. Additional S–L, S–Y, and L–Y paths allow incomplete mediation; a good fit for the sequential component alone would not establish that all structural or literacy associations operate through it [19,21].

Moderation describes variation in a relationship rather than an intermediate causal step. External access conditions A, defined by availability and affordability, are proposed to moderate L–B; care quality Q is a candidate moderator of B–Y. A moderation test therefore concerns a prespecified L-by-A or B-by-Q interaction and its uncertainty. Structural exposures are not reclassified as mediators: educational resources are proposed to mediate a specified socioeconomic relationship, whereas defined service conditions are proposed to moderate specified downstream relationships. These roles can coexist within a broader structural context but must be distinguished in each analysis [11,12,13,21].

The core model does not require every possible extension to be estimated simultaneously. Self-efficacy may be added as a mediator of the L–B relationship, and independently measured social support may be added as a moderator of that relationship, with prespecified hypotheses and adequate data. These are candidate extensions, not established mechanisms or substitutions for the access interaction in H3. Service use and disease may also influence later L; these feedback paths require time-indexed repeated measures and are not part of a cross-sectional causal mediation claim.

Socioeconomic position should be defined independently of E, and measures of A and Q should be distinct from OHL, realised attendance, and outcomes. Candidate indicators include income or occupation for S, schooling and independently assessed learning resources for E, domain-mapped OHL tasks for L, repeated assessments of practices or service records for B, and standardised clinical and quality-of-life measures for Y. Service availability, travel time, and affordability can be used to assess A; independently assessed continuity of care or the quality of preventive communication can be used to assess Q. H1–H5 concern selected core propositions rather than every arrow in Figure 3 and Figure 4. Testing each indirect effect requires temporal ordering and explicit confounding assumptions.

For example, a study of childhood caries could assess caregiver competencies, household educational resources, and independently measured service barriers. A factorial or cluster-randomised design could compare literacy support, improved service access, their combination, and usual care. This would test whether contextual support enhances the effect of literacy support on preventive attendance and whether changes in caregiver OHL precede behavioural and clinical changes. The caregiver, child, service, and community units of analysis must be distinguished. Such a study could identify where the proposed pathway fails, rather than treating any benefit from a multicomponent programme as validation of the entire framework (Figure 4).

### 3.7. Testable Hypotheses

The following hypotheses are proposed for future studies; none has been tested in the present manuscript. Their directions, primary outcomes, covariates, timing, and smallest effects of interest should be prespecified. A precisely estimated null association or an association in the opposite direction in an adequately powered, methodologically sound study would challenge the corresponding proposition; an imprecise estimate would remain inconclusive.

**H1 (Educational mediation).**  *Socioeconomic advantage (S), defined independently of schooling, will be associated with higher subsequent OHL (L) partly through greater educational and cultural resources (E). Repeated measures should test the S–E–L indirect relationship, accounting for baseline L and relevant confounding. A negligible indirect association would weaken this proposed mechanism, without excluding additional structural pathways.*

**H2 (Competency-specific contribution).**  *Interactive and critical OHL will predict subsequent preventive attendance or sustained self-care beyond functional OHL, schooling, baseline behaviour, and relevant covariates. The hypothesis is not supported if these domains add no meaningful, reproducible information. Preventive attendance should be distinguished from symptom-driven visits.*

**H3 (Contextual moderation).**  *The positive association between OHL and subsequent preventive attendance will be stronger where independently measured dental service availability and affordability are greater. This can be tested through a prespecified OHL-by-access interaction, with access measured at the relevant person or service level. A precisely estimated null interaction or a reproducible association in the opposite direction would require revision of this proposed enabling mechanism.*

**H4 (Behavioural mediation).**  *Higher OHL will predict a lower incidence of untreated caries during follow-up partly through sustained preventive behaviours, conditional on baseline disease and relevant confounders. A longitudinal design should separate competency assessment, behavioural follow-up, and clinical assessment. Absence of the predicted indirect relationship would challenge this pathway; effects on other outcomes would require separate hypotheses.*

**H5 (Combined intervention).**  *For a prespecified preventive attendance outcome and follow-up period, combined literacy support and improved service access will produce a larger absolute improvement than either component alone. A factorial trial can estimate each component and their interaction; superiority of the combination does not by itself imply synergy. Failure of the combination to improve attendance beyond the components would challenge this intervention proposition.*

## 4. Discussion

MELSO is proposed as an oral-health-specific specification of established theoretical relationships. Its contribution should be assessed against what existing frameworks already explain. Nutbeam distinguishes functional, interactive, and critical literacy [1,2]; Sørensen et al. integrate information-processing competencies with social and personal antecedents and health consequences [3]; Dahlgren and Whitehead situate health within layered social determinants [5]. Paasche-Orlow and Wolf specify pathways involving access, patient–provider interaction, and self-care [21], while HeLD provides a multidimensional measurement approach in dentistry [24]. MELSO does not introduce these concepts or the proposition that health literacy is socially conditioned.

MELSO’s proposed distinguishing feature is the explicit mediating role of E in the relationship between separately defined S and L, coupled with access-related moderation of L–B. In Sørensen et al.’s integrated model, social and personal antecedents, information-processing competencies, and health consequences are already connected [3]. MELSO retains these ideas but specifies an educational-resource mediator and a specified interaction in the oral health context for testing; it does not claim that social contextualisation is new.

The model proposed by Paasche-Orlow and Wolf is a relevant explanatory comparator because it links literacy with access and utilisation, patient–provider interactions, and self-care [21]. MELSO distinguishes external availability and affordability from realised use and assigns external access a specified moderating role in the L–B relationship, alongside the upstream E mediator. These are proposed analytical choices, not evidence of greater explanatory power. The application of literacy-sensitive communication to oral health has also been tested in a dental trial based on an existing conceptual model [28]; MELSO does not introduce this intervention approach. Its additional question is whether educational resources and service constraints jointly account for variation in intervention uptake or effects.

For example, if interactive or critical competencies predict later preventive action beyond functional literacy and schooling, and if that relationship is stronger where services are affordable and available, the results would support a distinction between acquiring competencies and being able to use them. Conversely, if the additional domains and interactions provide no reproducible explanatory or predictive improvement, the more elaborate MELSO specification would require simplification or revision. This capacity to discriminate between competing explanations is the intended route from conceptual integration to new knowledge.

### 4.1. Implications for Research

Empirical research should evaluate the measurement model and the proposed structural relationships separately. A valid OHL scale would support measurement of a core construct; it would not validate the full five-level framework. Equally, an association between an OHL score and disease would not establish the proposed educational mediation or contextual moderation.

A prospective multilevel study could measure socioeconomic conditions, service characteristics, and baseline disease, assess educational resources and OHL before repeated behavioural assessments, and obtain subsequent clinical and patient-reported outcomes. Age, language, prior care experience, and baseline health should be considered in a prespecified causal diagram; adjustment should be guided by the estimand and should not indiscriminately control for mediators. Multilevel models can separate person-level from service- or area-level associations [22]. Mediated pathways and interactions can then be estimated with uncertainty intervals and sensitivity analyses, accounting for clustering, missing data, and measurement error.

Cross-sectional studies can evaluate feasibility, construct structure, and associations compatible with MELSO, but cannot establish temporal ordering or causal mediation. Longitudinal observation improves temporal assessment without eliminating unmeasured confounding or reverse causation: dental disease and prior care can also alter OHL. Causal interpretations of indirect effects require explicit assumptions about confounding and timing; structural equation model fit alone does not establish those assumptions.

Incremental value should be assessed using prespecified comparisons. A baseline model could include socioeconomic position, schooling, functional OHL, baseline disease, and relevant covariates; extended models could add interactive and critical OHL, educational-resource pathways, and access interactions. Comparisons should use the same outcomes and samples, appropriate fit criteria, and out-of-sample predictive performance where prediction is intended. A direct-effects model and plausible alternative temporal structures should also be considered. A good fit for the full model is insufficient if simpler explanations perform equally well [21,22,23].

#### Measurement Development and Validation

Existing instruments should first be mapped to the intended competency dimensions, population, language, and setting [8,9,24]. Table 3 summarises their potential contributions and limitations; it is a conceptual mapping, not evidence that these measures validate MELSO. The illustrative tasks require development and validation before use.

HeLD also includes access, economic barriers, support, receptivity, and utilisation domains [24]. Its total score may therefore combine capabilities with contextual constraints. If access is modelled separately in MELSO, overlapping items must be identified to avoid circular associations. Any subscale selection or adaptation requires further validation; original scoring should not be altered without justification.

If gaps remain, patients from diverse educational and socioeconomic backgrounds, dental professionals, and measurement specialists should contribute to construct definitions and item generation. Cognitive interviews should assess comprehension, relevance, and cultural appropriateness before piloting. Performance tasks can complement self-reported ability, with standardised scoring and assessment of agreement between raters where judgement is required. Items should assess capability rather than simply reproduce attendance, affordability, or disease measures used elsewhere in the model.

Exploratory work and confirmatory analyses using independent data should compare plausible one-factor, correlated-domain, and hierarchical structures without assuming that the three OHL domains form a single developmental hierarchy. Validation should examine content and structural validity, internal consistency where appropriate, test–retest reliability, convergent and discriminant validity, and prediction of later behaviours beyond existing measures. Measurement invariance or differential item functioning should be evaluated across educational, socioeconomic, language, and age groups before comparing scores. Responsiveness requires intervention or longitudinal data. Sample sizes should be justified for the intended measurement model and subgroup analyses.

The five framework levels should not automatically be collapsed into a single MELSO score: structural exposures, educational resources, competencies, behaviours, and outcomes have different explanatory roles. Educational and cultural capital may require separate observed indicators or a justified formative specification rather than an assumed reflective scale. Validating an OHL instrument and evaluating the framework’s mediation and moderation hypotheses are complementary but separate tasks.

### 4.2. Implications for Oral Health Education

The educational implications follow from the proposed E–L pathway within H1 and the competency-specific contribution to B in H2. For example, programmes could assess whether general literacy and learning resources influence gains from oral health education, and then adapt information, dialogue, and appraisal tasks to measured competency profiles [1,14,15,19]. These educational approaches predate MELSO; the framework’s proposed contribution is to evaluate them alongside the upstream resources and downstream access conditions specified in the pathway.

An educational study could measure E and baseline L, assess changes in functional, interactive, and critical dimensions, and subsequently assess B while measuring A. This would distinguish limited competency gains from difficulty translating improved L into action, as anticipated by H3. Social disadvantage should not be used to presume a literacy profile or restrict opportunities for critical learning [19,26].

Programme activities may include interpreting preventive advice, asking questions, appraising online claims, and comparing treatment alternatives. Their content and feasibility should be developed with participants and evaluated against the specified L and B outcomes; the three dimensions are not successive course stages [1,3,26].

Across school, community, and dental settings, the life-course proposal supports repeated assessment of changing learning and service opportunities rather than a single fixed literacy classification. Whether such an approach improves the maintenance of competencies or narrows inequalities remains to be tested [16,19].

### 4.3. Implications for Clinical Practice

Clinical implications derive from the L–B relationship and its proposed moderation by A, rather than from an assumption that literacy alone determines adherence. Clinicians could examine whether comprehension and communication needs coexist with travel, cost, or appointment barriers. These should be measured separately to distinguish capability from opportunity [21,26].

Literacy-sensitive communication and teach-back are existing practices, including in dental research [28]. A MELSO-informed evaluation could assess changes in relevant L dimensions, subsequent B, and variation by independently measured A. This tests a proposed mechanism and boundary condition; it does not imply that the framework itself improves the effectiveness of established communication methods.

The proposed moderation of B–Y by Q suggests evaluating whether similar preventive practices are associated with different outcomes under different care conditions. Service evaluations should define Q independently of patient literacy and outcome ratings and test the interaction while accounting for baseline disease. Health-literate service design is supported by existing literature; its specific role in MELSO remains hypothetical [19,26].

### 4.4. Implications for Public Policy and Health Equity

Policy implications follow both the proposed structural-to-outcome path and the proposed moderation of L–B by A. Coverage, service availability, and affordability may influence oral health beyond changes in individual competencies, consistent with upstream approaches to oral health inequality [11,12,13]. MELSO identifies these as separate pathways so that policy evaluation need not attribute every social gradient to literacy.

For example, an expansion in dental coverage could be evaluated by examining changes in A, preventive B, and Y, and by testing whether the L–B association varies before and after implementation. Education and learning-resource policies address a different proposed pathway, S–E–L [14,15,19]. Neither relationship should be treated as confirmed by the framework.

H5 provides a rationale for comparing literacy support, improved access, and their combination. Such comparisons can assess whether combined action improves uptake and outcomes across socioeconomic groups, rather than presuming that a multicomponent policy reduces inequality. A larger combined effect would not itself establish synergy or validate the full model [11,19,26].

These implications are pathway-linked research and evaluation proposals. The proposed contribution is coordinated measurement of educational mediation, competency differences, and contextual moderation; improved effectiveness or equity relative to existing approaches has not been demonstrated.

### 4.5. Future Research Directions

Future research should first evaluate the construct boundaries and hypotheses specified in Section 3.7 and Section 4.1. Feasibility studies can establish whether distinct educational resources, OHL competencies, access conditions, and behavioural outcomes can be measured without substantial overlap. Longitudinal studies and independent replication should then examine the proposed pathways across socioeconomic groups and service settings.

Measurement development should follow the staged programme in Section Measurement Development and Validation, beginning with the suitability of existing instruments [8,9,24]. A new MELSO-informed OHL instrument is justified only if important competency domains remain inadequately represented. Its development would not itself establish the validity of the structural framework.

Intervention research should compare educational support with contextual support and their combination, as illustrated in Section 3.6. Studies should examine the intended intermediate changes, implementation, differential uptake, and clinical outcomes, including whether benefits reach disadvantaged groups. Literacy gains without behavioural change would warrant investigation of service barriers and alternative mechanisms, rather than being interpreted as intervention failure at the individual level [25,26].

Finally, the framework encourages comparative and international research to explore how variations in education systems, dental care organisation, and social policies influence oral health literacy trajectories. Such research could contribute to a fuller understanding of oral health inequalities across diverse sociopolitical contexts.

### 4.6. Scope and Limitations

MELSO remains an unvalidated conceptual proposal. Its purposive narrative approach and targeted revision search do not support exhaustive novelty claims or an evidence-based estimate of the effects along each pathway. Educational resources, OHL, perceived access, and digital health literacy may overlap, requiring item mapping and discriminant-validity assessment. The life-course component identifies changing opportunities but lacks validated developmental trajectories, and the three literacy dimensions are not fixed stages.

Figure 3 and Figure 4 simplify potentially reciprocal relationships: earlier care and disease may alter later OHL and behaviour. These feedback propositions, the additional structural paths, and optional self-efficacy and social-support extensions require separate hypotheses, temporal data, and confounding assumptions. Service access and quality must not be counted simultaneously as competencies and contextual predictors. No findings are presented that establish model validity, superior performance, or effects on clinical outcomes or equity.

## 5. Conclusions

MELSO proposes an oral-health-specific specification in which educational and cultural resources may mediate the relationship between socioeconomic position and OHL, while access to care may moderate the translation of OHL into preventive behaviour. These paired propositions constitute its proposed distinguishing feature within established health literacy and social determinants perspectives.

The five framework levels organise constructs rather than developmental stages; functional, interactive, and critical OHL are interrelated dimensions with potentially uneven profiles. The hypotheses, candidate measures, and revised diagrams suggest ways to examine the proposed relationships and possible feedback between them over time.

MELSO has not been empirically validated, and its life-course mechanisms, measurement boundaries, and added explanatory value remain uncertain. Future studies must determine whether its predictions are measurable and reproducible and whether the specification adds value beyond existing models. Clinical usefulness and effects on oral health equity cannot yet be inferred.

## Figures and Tables

**Figure 1:**
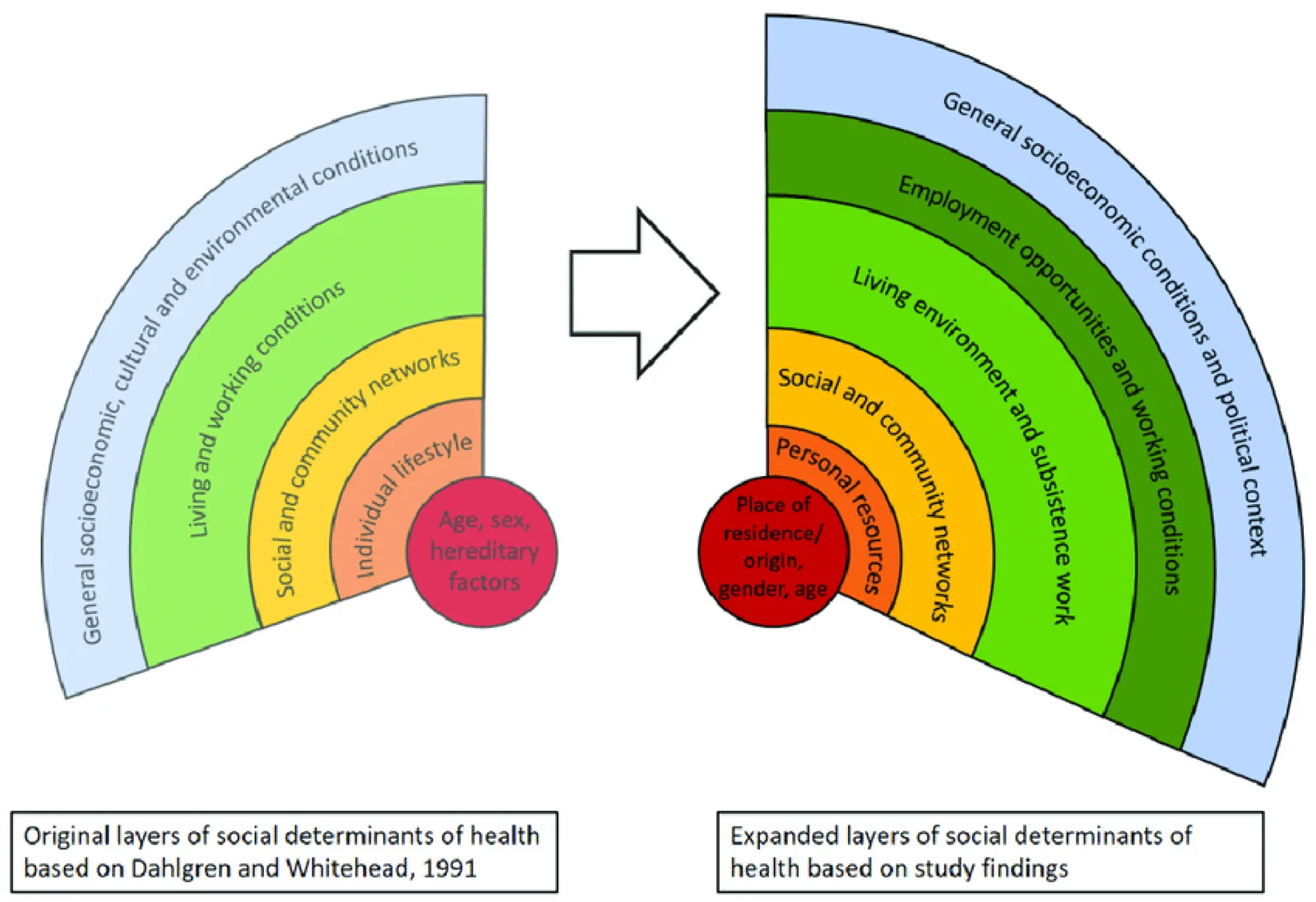
Social determinants of health model, adapted and conceptually expanded to illustrate structural and contextual influences relevant to oral health literacy [5].

**Figure 2:**
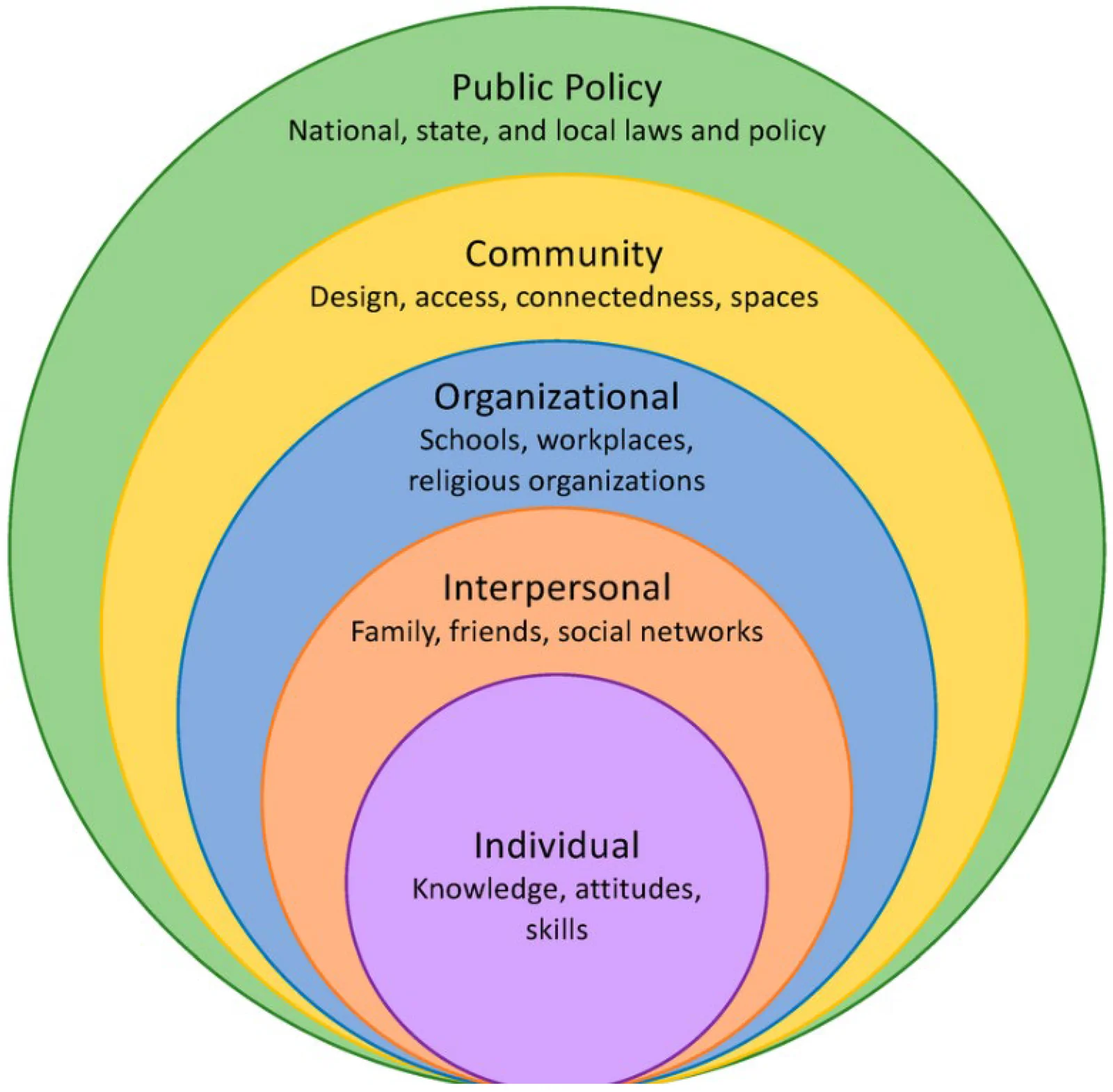
Ecological model of multilevel influences on health, ranging from individual factors to interpersonal, organisational, community, and public-policy contexts [16].

**Figure 3:**
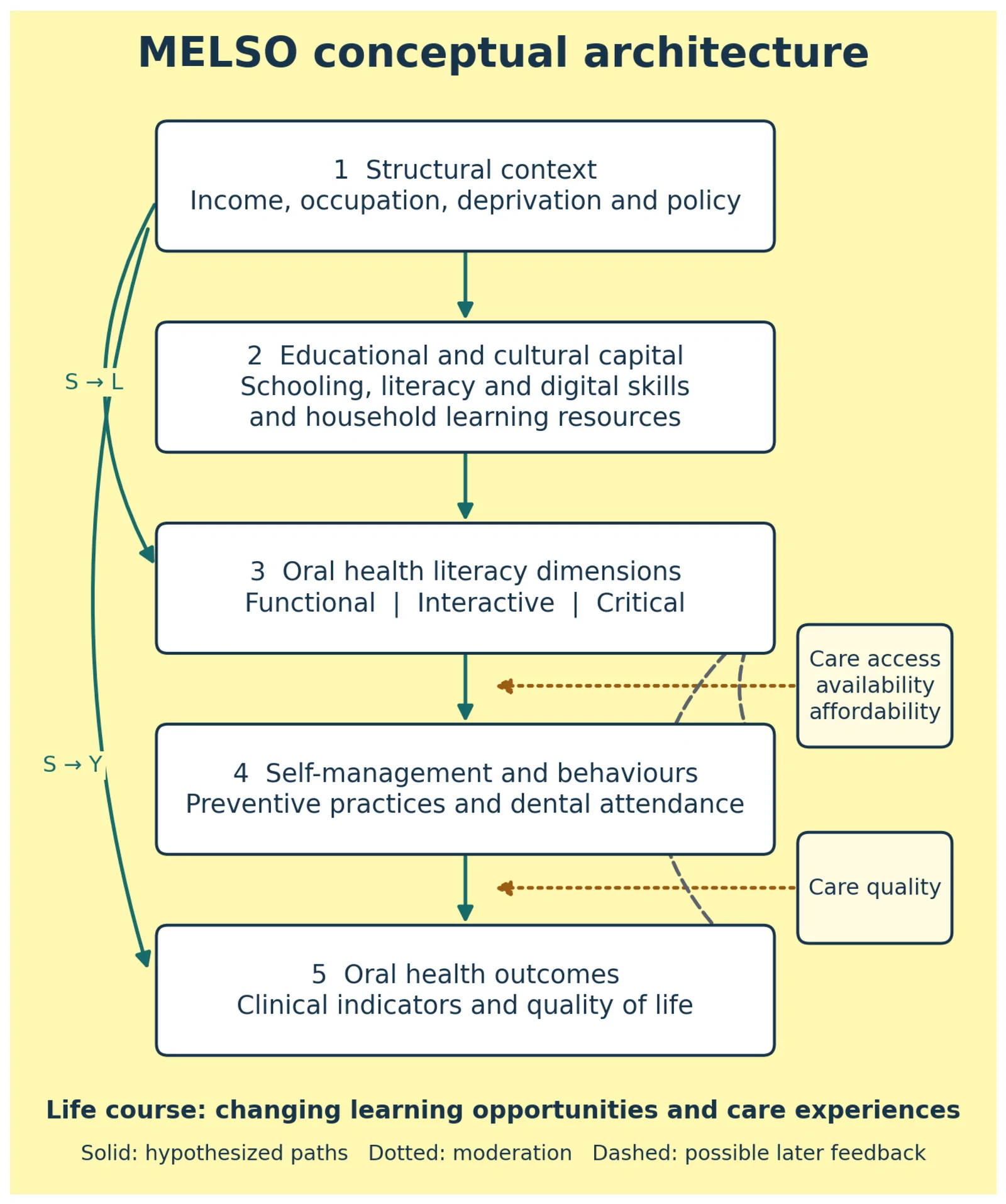
Revised MELSO architecture across the life course. Framework levels differ from literacy dimensions. Solid arrows show proposed paths, including structural associations outside the educational pathway (S to L) and literacy–behaviour pathway (S to Y). Dotted connectors indicate candidate moderation; dashed arrows indicate possible later feedback to OHL. Changing life-course opportunities do not imply fixed stages. S denotes structural context, L denotes OHL, and Y denotes outcomes. All relationships are hypothetical; yellow identifies the revised figure.

**Table 3:** Candidate coverage of MELSO literacy dimensions.

Dimension	Candidate Assessment	Coverage and Limits
Functional	REALD-30 [8]; OHLI [9]	REALD-30 assesses dental word recognition, not comprehension. OHLI assesses reading comprehension and numeracy. Neither assesses interactive or critical competence.
Interactive	HeLD communication and understanding domains [24]; consultation tasks	HeLD provides relevant self-reported abilities but its domains are not validated equivalents of the MELSO dimensions. Tasks could assess asking questions, clarifying advice, and discussing a care plan.
Critical	Appraisal and decision tasks requiring validation	Tasks could assess source credibility, interpretation of treatment benefits and harms, and reasoned decisions under social constraints. REALD-30, OHLI, and a HeLD total score should not be assumed to establish this domain.

## Data Availability

No new data were created or analysed in this study. Data sharing is not applicable to this article.
